# LINC00346 Sponges miR-30c-2-3p to Promote the Development of Lung Adenocarcinoma by Targeting MYBL2 and Regulating CELL CYCLE Signaling Pathway

**DOI:** 10.3389/fonc.2021.687208

**Published:** 2021-09-22

**Authors:** Qian Xu, Zhenwu Xu, Kai Zhu, Jinlan Lin, Bo Ye

**Affiliations:** ^1^ Department of Oncology Medicine, Fujian Medical University Union Hospital, Fuzhou, China; ^2^ Department of Thoracic Medical Oncology, Fujian Medical University Cancer Hospital, Fujian Cancer Hospital, Fuzhou, China; ^3^ Department of Thoracic Surgery, Affiliated Hangzhou Chest Hospital, Zhejiang University School of Medicine, Hangzhou, China

**Keywords:** lung adenocarcinoma, LINC00346, cell cycle, proliferation, metastasis

## Abstract

**Background:**

LINC00346 has recently been reported to regulate the development of several cancer types, but its biological functions and underlying mechanisms in lung adenocarcinoma (LUAD) have not been elucidated. The purpose of this study was to investigate the molecular mechanism of LINC00346 in the progression of LUAD.

**Methods:**

Bioinformatics was performed to find the target lncRNA, miRNA and mRNA, and the binding relationship between the target genes was verified by dual luciferase reporter gene and RIP assays. Fluorescence *in situ* hybridization was used to detect the location of LINC00346 in LUAD tissues. The expressions of LINC00346, miR-30c-2-3p and MYBL2 in each group were detected by qRT-PCR, and western blot was performed to detect expressions of MYBL2 and CELL CYCLE related proteins. Proliferation, metastasis, apoptosis and cell cycle of LUAD cells were detected by CCK-8, colony formation, Transwell and flow cytometry assays, respectively. Mouse xenograft models were established to further determine the effects of LINC00346 on LUAD tumor growth *in vivo*.

**Results:**

LINC00346 was upregulated in LUAD tissues and cells and was mainly localized in the cytoplasm. Knockdown of LINC00346 inhibited tumor growth *in vivo*, proliferation, metastasis and cell cycle progression, while induced apoptosis. LINC00346 sponged miR-30c-2-3 by targeting MYBL2 and regulating CELL CYCLE signaling pathway. Inhibiting miR-30c-2-3p or overexpressing MYBL2 could reverse the inhibitory effect of LINC00346 knockdown on LUAD process.

**Conclusions:**

LINC00346 as a ceRNA played a carcinogenic role in the development of LUAD *via* miR-30c-2-3p/MYBL2 axis regulating the CELL CYCLE signaling pathway. The study generally elucidated the mechanism by which LINC00346 regulated the development of LUAD, providing new ideas for the diagnosis and treatment of LUAD guided by lncRNA.

## Introduction

Lung cancer is the disease characterized by highest morbidity and mortality, accounting for nearly one fifth of cancer deaths (1). Lung cancer is divided into small cell lung cancer and non-small cell lung cancer (NSCLC), among which lung adenocarcinoma (LUAD) is the main histological subtype of NSCLC (2, 3). Despite improvements in early diagnosis and treatment, along with reports of several major oncogenic drivers including EGFR mutations, KRAS and ALK rearrangements (4), the prognosis of LUAD remains poor (5). The main reason for the poor prognosis is the lack of understanding of the biological mechanism associated with LUAD, which limits the improvement of treatment effect. Therefore, it is imperative that the pathogenesis of LUAD progression needs to be further clarified, so as to develop new treatment methods and improve the clinical outcome of LUAD patients.

Abundant evidence indicates that long non-coding RNAs (lncRNAs) are involved in the regulation of various biological and pathological processes and play a key role in the development of cancer (6). As oncogenes or tumor suppressor genes, lncRNAs are involved in the development of cancer, such as cell proliferation, metabolism, migration, invasion, cell cycle, apoptosis and autophagy (7). For example, lncRNA HOXA11-AS as a ceRNA promotes cisplatin resistance in LUAD cells through the miR-454-3p/Stat3 axis (8). LncRNA SBF2-AS1/miR-363-3p/E2F1 axis can promote the tumorigenesis of LUAD (9). CAR10 induces epithelial-mesenchymal transition (EMT) by directly binding with miR-30 and miR-203 along with regulating the expression of SNAI1 and SNAI2, ultimately promoting the metastasis of LUAD cells (10). LncRNA LOXL1-AS1 promotes cellular progression in LUAD by sponging miR-423-5p and targeting MYBL2 (11). Although some lncRNAs regulating the occurrence and development of LUAD have been found, the biological functions and potential mechanisms of most lncRNAs in LUAD remain to be elucidated.

Recent studies have found that LINC00346 is abnormally expressed in a variety of cancers as an oncogenic gene. LINC00346 is an intergenic lncRNA with a length of 6322 base pairs, and is located on chromosome 13q34 (12). LINC00346 is highly expressed in hepatocellular carcinoma (HCC) and promotes cell proliferation, migration, and invasion (13). It also induces tumor growth in HCC by activating the JAK-STAT3 pathway (12). Up-regulated LINC00346 promotes the development of pancreatic cancer by activating c-Myc (14). While knockdown of LINC00346 suppresses the proliferation and migration of bladder cancer cells, and induces cell cycle arrest and apoptosis (15). These researches suggest that LINC00346 may be a potential biomarker and target for cancer diagnosis and treatment. However, little is known about the regulatory mechanism of LINC00346 in LUAD.

In this study, we explored the expression pattern of LINC00346 in LUAD, and investigated the biological role and the possible molecular mechanism of LINC00346 in regulating the malignant phenotype of LUAD cells. The research contributes to a better understanding of the carcinogenetic effect of LINC00346 and tumor progression, providing potential targets for the diagnosis and treatment of LUAD.

## Materials and Methods

### Bioinformatics Analysis

Mature miRNA (Normal: n=46, Tumor: n=521), mRNA and lncRNA (Normal: n=59, Tumor: n=535) expression profiles and clinical data of LUAD were downloaded from The Cancer Genome Atlas (TCGA) database. Differential analysis was conducted on lncRNAs in normal and tumor groups using the ‘edgeR’ package with the threshold of |logFC|>1 and padj<0.05 to obtain differentially expressed lncRNAs (DElncRNAs). The miRcode database was used to determine DElncRNA- DEmiRNA pairs, and target lncRNA was identified by the survival analysis. The downstream miRNAs of the target lncRNA were predicted through the lncBase database, and intersected with DEmiRNAs (|logFC|>1, padj<0.05) to determine the target miRNA. Target genes of miRNA were predicted by miRDB and TargetScan databases, and the target mRNA was identified by intersection between predicted mRNAs and DEmRNAs (|logFC|>2, padj<0.05). Clinical staging analysis was performed on target mRNA, and pathway enrichment analysis was conducted on target lncRNA, miRNA and mRNA by GSEA.

### Patients and Tissue Samples

A total of 40 pairs of LUAD and para-cancerous tissue samples were obtained from patients undergoing surgical resection of tumors in Hangzhou Red Cross Hospital from January 2018 to December 2019. The samples were diagnosed as LUAD by histopathological evaluation and frozen immediately in a liquid nitrogen container. The patients had not received radiotherapy or chemotherapy before surgery, acquired information of sample collection and signed informed consent. The project was approved by the research ethics committee of Hangzhou Red Cross Hospital.

### Cell Culture

Human normal bronchial epithelial cell line BEAS-2B (ATCC ^®^ CRL-9609^™^) and LUAD cell lines NCI-H1395 (ATCC ^®^ CRL-5868^™^), NCI-H1975 (ATCC ^®^ CRL-5908^™^), A549 (ATCC ^®^ CCL-185^™^) and H157 (ATCC ^®^ CCL-185^™^) were purchased from the American Type Culture Collection (ATCC; Manassas, VA). Cells were cultured in Dulbecco’s Modified Eagle Medium (DMEM) supplemented with 10% fetal bovine serum (FBS; Corning, Corning, NY) at 37°C, containing 5% CO_2_.

### Plasmid Construction and Cell Transfection

The lentivirus-coated sh-LINC00346 vector and corresponding negative control were synthesized by Sangon Biotech (Shanghai, China). miR-30c-2-3p inhibitor/miR-30c-2-3p-mimic and their negative controls along with MYBL2 overexpression vector and corresponding control were purchased from Dharmacon (Lafayette, CO, USA). Lipofectamine 2000 (Invitrogen, Carlsbad, CA, USA) was used for cell transfection according to the manufacturer’s instructions. Cells were collected 48 h after transfection.

### qRT-PCR

Total RNA was isolated from cells or tissues using TRIzol reagent (Invitrogen). The quantity and quality of RNA were assessed by Nanodrop ND-2000 spectrophotometer (Thermo Scientific^™^, USA). 2 μg of total RNA was reversed into cDNA using the RevertAid First Strand cDNA Synthesis Kit (Thermo Scientific^™^, USA) to evaluate the expression of LINC00346 and MYBL2. 2 μg of total RNA was reversed into miRNA cDNA using M-MLV Reverse Transcriptase (Invitrogen) for the expression analysis of miR-30c-2-3p. qRT-PCR was performed on the StepOne real-time PCR system (Thermo Fisher Scientific) using SYBR Green PCR kit (Takara Bio, Otsu, Japan). The relative gene expression standardized by GAPDH and U6 was calculated by 2^-ΔΔCt^ method. Primer sequences were listed in **Table 1**.

**Table 1:** Primer sequence for qRT-PCR.

Gene	Forward (5’-3’)	Reverse (5’-3’)
**LINC00346**	CACCATGTTGGCCAGGCTGGT	GGCCAAAGAGTGACCATCATC
**MYBL2**	CTTGAGCGAGTCCAAAGACTG	AGTTGGTCAGAAGACTTCCCT-
**GAPDH**	CCCATCACCATCTTCCAGGAG	CTTCTCCATGGTGGTGAAGACG
**miR-30c-2-3p**	GGCTGGGAGAAGGCTGTT	AGTGCGTGTCGTGGAGT
**U6**	CTCGCTTCGGCAGCACA	AACGCTTCACGAATTTGCGT

### Western Blot

Cells were lysed in the lysis buffer and the protein concentration was measured using the BCA protein assay kit (Thermo Fisher Scientific). The 10 μg proteins were separated by sodium dodecyl sulfate polyacrylamide gel electrophoresis (SDS-PAGE) and transferred onto the polyvinylidene fluoride (PVDF) membranes (Millipore). The membranes were blocked with 5% skim milk for 2 h and incubated with rabbit anti-MYBL2 (1:1000, ab76009, Abcam, Cambridge, UK), cyclin A2 (1:10000, ab32386, Abcam, Cambridge, UK), cyclin B1 (1:20000, ab32053). Abcam, Cambridge, UK), GAPDH (1:10000, ab180630, Abcam, UK) and Plk1 (1:1000, ab155095, Abcam, Cambridge, UK) overnight at 4°C. Then the membranes were cultured with secondary antibody goat anti-rabbit IgG H&L (ab205718, Abcam, Cambridge, UK) for 1 h at room temperature. Finally, the electrochemiluminescence kit (ECL; Pierce Biotechnology) was used for protein development.

### Fluorescence *In Situ* Hybridization (FISH)

LINC00346 FISH probe was labeled with 5-carboxyl fluorescein. The tissues were digested by protease K, denatured by formamide, and hybridized with the LINC00346 probe overnight at 42°C. Then tissues were stained with DAPI (4’,6‐diamidino‐2‐phenylindole). The samples were analyzed and photographed under a laser scanning confocal microscope (LSM700, Carl Zeiss, Germany).

### CCK-8 Assay

Cell proliferation was detected using Cell Counting kit-8 (Beyotime, Shanghai, China). Cells were inoculated into 96-well plates at a concentration of 5×10^3^ cells/well. After incubation for 0 h, 24 h, 48 h, 72 h and 96 h, respectively, 10 μl CCK-8 solution was added to each well and cells were cultured for another 2 h at 37°C. The absorbance of each well at 450 nm wavelength was measured by a microplate reader (Bio-Rad, Hercules, CA, USA).

### Colony Formation Assay

48 h after transfection, the cells (1×10^3^ per well) were inoculated into 6-well plates and cultured in complete medium for 2 weeks until obvious colonies were observed. Cell colonies were fixed with 4% paraformaldehyde for 30 min and stained with 0.5% crystal violet at room temperature for 30 min. Each well was washed with sterile water to remove residual crystal violet. The number of colonies (more than 50 cells/colony) was calculated.

### Transwell Assay

Migration and invasion measurements were conducted using Transwell inserts (8 μm pore size; Costar, Cambridge, MA, USA). Cells were inoculated into the upper chambers without or precoated with Matrigel (BD, Franklin Lakes, USA) for migration or invasion analysis, and 600 μl medium containing 10% FBS was placed in the lower chambers. After incubation at 37°C with 5% CO_2_ for 24 h, the cells in the upper chambers were removed with cotton swabs, and the migrated or invaded cells in the lower chambers were fixed with methanol, stained with 0.5% crystal violet and washed with PBS (Gibco; Thermo Fisher Scientific, Inc.). Then cells were counted under a microscope (Zeiss, Germany) at 100× magnification.

### Flow Cytometry (FCM)

In order to analyze apoptosis, the cells were digested with trypsin and then resuspended in the binding buffer containing Annexin V-FITC (BD Biosciences, San Jose, CA, USA) and propidium iodide (PI; BD Biosciences) for 15 min in the dark. The stained cells were analyzed using FCM (BD Biosciences).

Cells were fixed with cold 70% ethanol for 12 h and incubated with PI (50 μg/mL) and RNase A (Sigma-Aldrich) for 30 min. Cell cycle distribution was analyzed by FCM.

### Dual-Luciferase Reporter Gene Assay

The 3’-UTR of LINC00346 or MYBL2 was amplified from human genomic DNA. These sequences were then subcloned into the pGL3 luciferase reporter vector (Promega, Madison, WI, USA), respectively. The potential binding sites paired with miR-30c-2-3p were mutated by using Quick-change site-directed mutagenicity kit (Agilent Technologies, Santa Clara, CA, USA). The wild type (wt) and mutant (mut) 3’-UTR of LINC00346 or MYBL2 along with either NC mimic or miR-194-5p mimic were co-transfected into LUAD cells, respectively. The luciferase activity of firefly and renilla luciferase was measured by Dual-Lucy Assay Kit (Promega, Madison, WI, USA) 48 h after transfection.

### RNA-Binding Protein Immunoprecipitation (RIP)

Magna RNA immunoprecipitation (RIP) kit (Millipore, Billerica, USA) was used for RIP detection. Transfected cells were re-suspended in the lysis buffer. 100 μl of whole cell extracts were incubated with Protein G agarose microbeads conjugated with Ago2 antibody (Abcam, Cambridge, UK) or IgG (Abcam, Cambridge, UK) overnight at 4°C. The immunoprecipitates were treated with DNA enzyme I and protease K at room temperature for 20 min. The co-precipitated RNA was recovered and analyzed by qRT-PCR.

### Animal Experiment

To confirm the role of LINC00346 in promoting LUAD cell proliferation *in vivo*, we constructed mouse xenograft tumor models. Ten male BALB/c nude mice [National 0Laboratory Animal Center (Beijing, China)](4 weeks old) were randomly divided into two groups (5 per group). LUAD cells transfected with sh-LINC00346 or sh-NC were washed with PBS and subcutaneously injected into the underarm area on either side of the nude mice. Tumor size was assessed weekly with caliper and the tumor volume was estimated by the following formula: volume = length × width^2^ × 0.5. The mice were euthanized after 5 weeks, and the tumors were isolated to measure the weight.

### Immunohistochemistry (IHC)

Immunostaining of paraffin-embedded xenograft tumor tissues was performed. IHC was performed by means of streptavidin coupled with peroxidase, with rabbit anti-MYBL2 antibody (1:100, ab76009, Abcam, Cambridge, UK) and Ki67 antibody (1:50, ab15580, Abcam, Cambridge, UK) as primary antibodies and goat anti-rabbit IgG H&L (ab205718, Abcam, Cambridge, UK) as secondary antibody. Non-immune serum was used to replace primary antibody as negative control. Sections were observed under a 400× or 200× microscope (ZEISS, Germany).

### Statistical Analysis

SPSS 22.0 (IBM Corp. Armonk, NY, USA) and GraphPad Prism 6.0 Software (GraphPad Inc., San Diego, CA, USA) were used for statistical analysis. We first analyzed the normal distribution of the measured data with F test. After comfirming that data meet the normal distribution, they were expressed as mean ± SD. The comparison between the two groups was analyzed by *t* test. ANOVA test was used for comparison among multiple groups. The Pearson correlation analysis was used to assess the correlation between different groups. *P*<0.05 indicated that the difference was statistically significant.

## Results

### LINC00346 Is Up-Regulated in LUAD Tissues and Cells, and Is Mainly Located in the Cytoplasm

**Figure 1:**
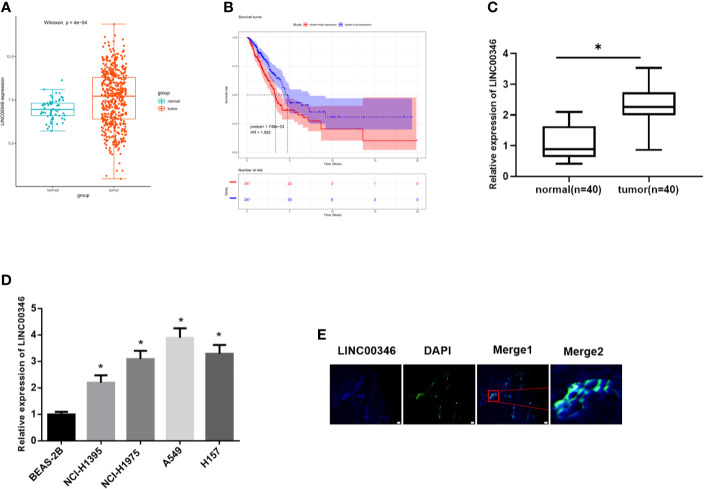
LINC00346 is up-regulated in LUAD tissues and cells, and is mainly located in the cytoplasm. **(A)** The expression of LINC00346 in normal (green) and tumor (red) samples; **(B)** The correlation between the expression level of LINC00346 (red for high-expression group and blue for low-expression group) and overall survival in LUAD patients was analyzed by Kaplan-Meier survival analysis; **(C)** The expression of LINC00346 in 40 pairs of clinical LUAD and para-cancerous tissues, and **(D)** in normal bronchial epithelial cell line and LUAD cell lines were measured by qRT-PCR; **(E)** FISH was used to detect the localization of LINC00346 in LUAD. *P < 0.05.

TCGA-LUAD data indicated that LINC00346 was highly expressed in LUAD tissues (**Figure 1A**), and the survival rate of patients with high expression of LINC00346 was significantly lower than that with low expression (**Figure 1B**). Furthermore, the results of qRT-PCR found that the expression of LINC00346 in clinical LUAD tissues (n=40) was significantly higher than that in adjacent normal tissues (n=40) (**Figure 1C**). In addition, we also detected the expression of LINC00346 in LUAD cells, and the results exhibited that, compared with BEAS-2B cell line, LINC00346 was remarkably up-regulated in LUAD cell lines NCI-H1395, NCI-H197, A549 and H157 (**Figure 1D**). The subcellular localization of lncRNAs is closely related to biological function and potential molecular mechanism (16). Therefore, we detected the subcellular localization of LINC00346 in the tumor tissues of LUAD patients by RNA-FISH. The results indicated that LINC00346 was mainly located in the cytoplasm, and a few were distributed in the nucleus (**Figure 1E**). Based on the above results, we believed that LINC00346 was up-regulated in both LUAD tissue and cells, and may play a role as ceRNA. In addition, LUAD cells NCI-H1395 and A549 had the most significant differential expression of LINC00346 compared with normal cell lines, so these two cell lines were selected for subsequent functional studies.

### LINC00346 Inhibits the Proliferation, Migration and Invasion of LUAD Cells and Promotes Apoptosis

**Figure 2:**
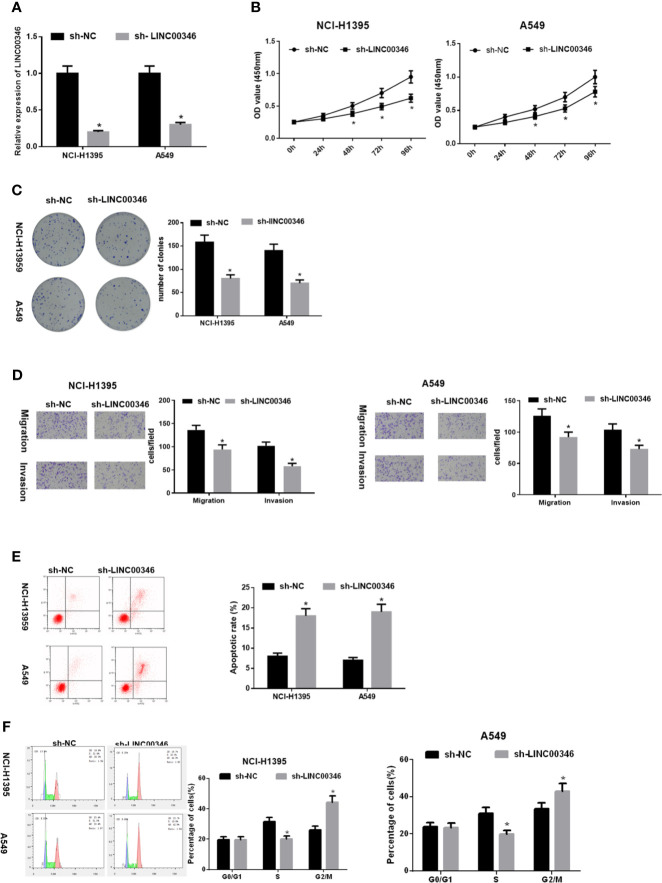
LINC00346 inhibits the proliferation, migration and invasion of LUAD cells and promotes apoptosis. After transfecting sh-LINC00346 into LUAD cells, **(A)** the expression of LINC00346 in NCI-H1395 and A549 cells, **(B)** LUAD cell proliferation, **(C)** colony formation ability, **(D)** migration and invasion, **(E)** apoptosis and **(F)** cell cycle distribution were detected by qRT-PCR, CCK-8, colony formation, Transwell and FCM, respectively. *P < 0.05.

To evaluate the biological role of LINC00346 in LUAD, the expression of LINC00346 was inhibited by transfecting sh-LINC00346 into NCI-H1395 cells and A549 cells (**Figure 2A**). The results of CCK-8 (**Figure 2B**), colony formation (**Figure 2C**) and Transwell (**Figure 2D**) assays displayed that knockdown of LINC00346 inhibited cell proliferation, migration and invasion. In addition, FCM with Annexin V-FITC/PI double staining was used to detect cell apoptosis and cycle changes. It was showed that knockdown of LINC00346 promoted cell apoptosis (**Figure 2E**) and significantly reduced the cell proportion in the S phase, blocked the cell cycle in the G2/M phase (**Figure 2F**). Therefore, silencing LINC00346 inhibited the proliferation and metastasis of LUAD cells and promoted apoptosis.

### LINC00346 Acts as a Molecular Sponge of miR-30c-2-3p

**Figure 3:**
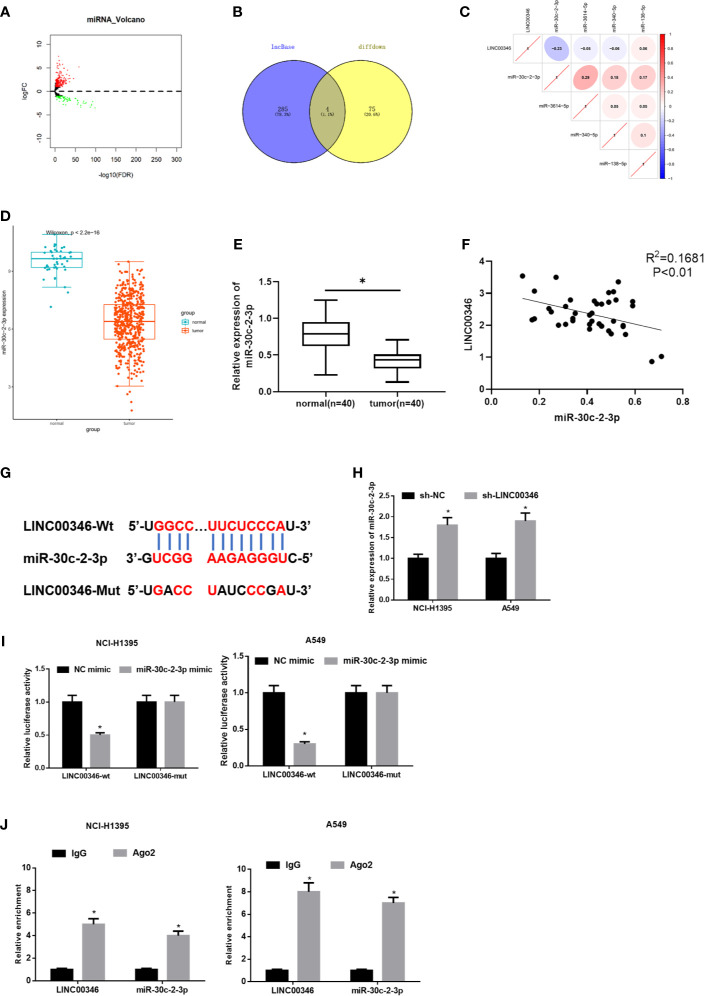
LINC00346 acts as a molecular sponge of miR-30c-2-3p. **(A)** Volcano plot of DEmiRNAs in TCGA-LUAD dataset; **(B)** The Venn diagram of predicted downstream miRNAs and down-regulated DEmiRNAs; **(C)** Pearson correlation analysis of LINC00346 and miR-30c-2-3p; **(D)** The expression of miR-30c-2-3p in TCGA-LUAD (green represents the normal samples and the red represents the tumor samples); **(E)** qRT-PCR was used to detect the expression of miR-30c-2-3p in 40 pairs of clinical samples; **(F)** Correlation analysis indicates a negative correlation between LINC00346 and miR-30c-2-3p expression in 40 LUAD tissue samples; **(G)** The targeted binding sequence of LINC00346 and miR-30c-2-3p was predicted by lncBase database; **(H)** Expression level of miR-30c-2-3p in NCI-H1395 cells and A549 cells after transfection with sh-LINC00346; **(I)** Dual-luciferase reporter assay verified the targeted binding relationship between miR-30c-2-3p and LINC00346 in LUAD cells; **(J)** The enrichment of miR-30c-2-3p and LINC00346 in Ago2 immunoprecipitation was detected by RIP. *P < 0.05.

In order to further verify the regulatory mechanism of LINC00346 in LUAD. Firstly, 299 differentially expressed miRNAs were obtained through differential analysis (**Figure 3A**). Then, we used lncBase database to conduct target prediction of LINC00346, and the predicted results were intersected with 79 down-regulated DEmiRNAs to obtain 4 DEmiRNAs bearing targeted binding sites with LINC00346 (**Figure 3B**). After Pearson correlation analysis between LINC00346 and the four miRNAs, miR-30c-2-3p with the highest correlation coefficient and a negative correlation with LINC00346 was selected for further study (**Figure 3C**). Differential analysis of miRNAs downloaded from TCGA-LUAD revealed that miR-30c-2-3p was extremely lowly expressed in LUAD (**Figure 3D**). Then, qRT-PCR was used to verify the above prediction results in 40 pairs of collected tissue samples. It was found that the expression of miR-30c-2-3p in LUAD tissue was significantly down-regulated (**Figure 3E**), and its expression level in LUAD tissue was significantly negatively correlated with the expression of LINC00346 (**Figure 3F**). LINC00346 was found to have complementary binding sites with miR-30c-2-3p through analysis using lncBase database (**Figure 3G**).

To confirm the above prediction, we firstly transfected sh-LINC00346 into NCI-H1395 and A549 cells to observe whether the miR-30c-2-3p level was affected by sh-LINC00346. The qRT-PCR results showed that the expression of miR-30c-2-3p was significantly up-regulated after cells were transfected with sh-LINC00346 (**Figure 3H**). LINC00346-wt and LINC00346-mut reporter vectors were constructed and dual luciferase reporter gene assay was performed. It was found that luciferase activity of the LINC00346-wt group was significantly reduced with miR-30c-2-3p mimic, while that of the LINC00346-mut group was not affected (**Figure 3I**). RIP assay indicated that after immunoprecipitation with Ago2 antibody, miR-30c-2-3p and LINC00346 were enriched in NCI-H1395 and A549 cells (**Figure 3J**), indicating a possible binding relationship between miR-30c-2-3p and LINC00346. In consequence, there was a direct interaction between LINC00346 and miR-30c-2-3p in LUAD.

### Inhibition of miR-30c-2-3p Reverses the Effects of LINC00346 Knockdown on Proliferation, Migration and Invasion of LUAD Cells

**Figure 4:**
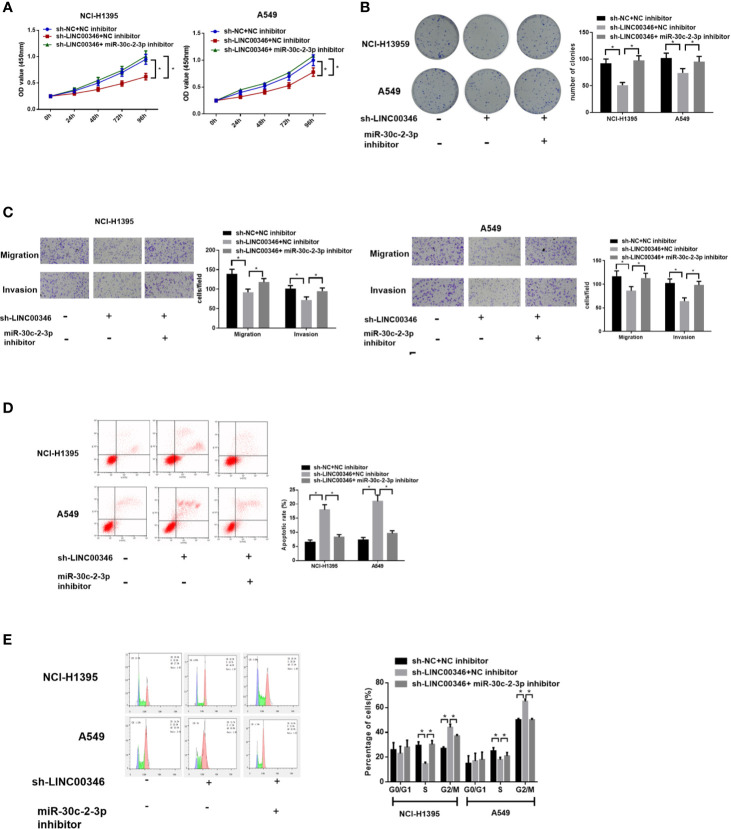
Inhibition of miR-30c-2-3p reverses the effects of LINC00346 knockdown on LUAD cells. Cell proliferation **(A)**, colony formation ability **(B)**, migration and invasion **(C)**, apoptosis and cell cycle distribution **(D, E)** of each group were analyzed by CCK-8, colony formation, Transwell and FCM assays, respectively. *P < 0.05.

To investigate whether LINC00346 regulates the malignant phenotype of LUAD cells by targeting miR-30c-2-3p, we transfected sh-NC+NC inhibitor, sh-LINC00346+NC inhibitor, sh-LINC00346+miR-30c-2-3p inhibitor into NCI-H1395 cells and A549 cells, respectively, and evaluated cell proliferation, migration, invasion, cell cycle, and apoptosis. It was observed that the knockdown of LINC00346 inhibited the proliferation, migration and invasion of LUAD cells (**Figures 4A–C**) as well as promoted cell apoptosis (**Figure 4D**) and reduced cell proportion in the S phase (**Figure 4E**), while the inhibition of miR-30c-2-3p could attenuate such effects. Therefore, inhibition of miR-30c-2-3p reversed the effects of LINC00346 knockdown on proliferation, migration and invasion of LUAD cells.

### LINC00346 as a ceRNA Regulates the CELL CYCLE Signaling Pathway Through miR-30c-2-3p and Targeting MYBL2

**Figure 5:**
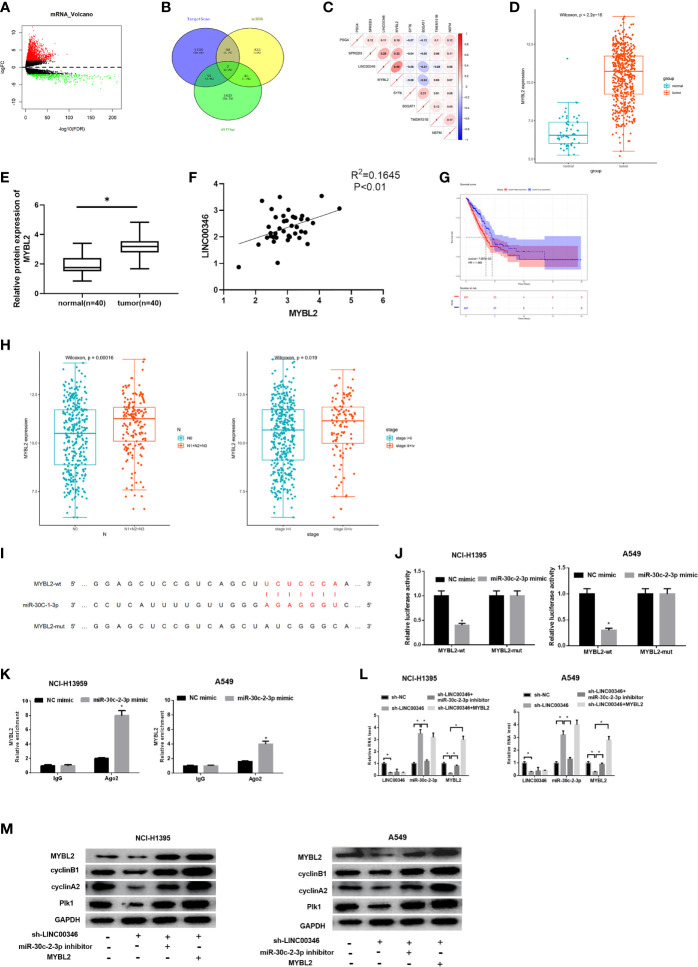
LINC00346 regulates CELL CYCLE signaling pathway by sponging miR-30c-2-3p and targeting MYBL2. **(A)** Volcano plot of DEmRNAs in TCGA-LUAD dataset; **(B)** Venn diagram of predicted mRNAs and up-regulated DEmRNAs; **(C)** Pearson correlation analysis of LINC00346 and MYBL2; **(D)** The boxplots of MYBL2 expression in normal (green) and tumor (red) samples; **(E)** qRT-PCR was used to detect the expression of MYBL2 mRNA in 40 pairs of clinical samples; **(F)** Correlation analysis indicates a highly positive correlation between LINC00346 and MYBL2 expression in 40 LUAD tissue samples; **(G)** Kaplan-Meier curves of MYBL2 expression in TCGA-LUAD (red for high-expression group and blue for low-expression group); **(H)** The expression of MYBL2 in different clinical stages and N stage of LUAD; **(I)** The binding sites of miR-30c-2-3p and MYBL2 predicted by TargetScan; **(J)** Dual-luciferase reporter gene assay was used to determine the targeted relationship between miR-30c-2-3p and MYBL2; **(K)** The enrichment of MYBL2 in Ago2 immunoprecipitation was detected by RIP; **(L)** qRT-PCR verified the expression of LINC00346, miR-30c-2-3p and MYBL2 in each treatment group; **(M)** Western blot was used to detect the expression of MYBL2 and cell cycle related proteins in each group. *P < 0.05.

Totally 2503 mRNAs differentially expressed in LUAD were obtained using TCGA database (**Figure 5A**). miRDB and TargetScan databases were used to predict the target genes of miR-30c-2-3p, and the predicted results were intersected with the 1,975 up-regulated DEmRNAs to obtain 7 DEmRNAs that had the targeted binding sites with miR-30c-2-3p (**Figure 5B**). Pearson correlation analysis of LINC00346 and these 7 mRNAs showed that LINC00346 was positively correlated with MYBL2 with the highest correlation coefficient (**Figure 5C**). MYBL2 was highly expressed in LUAD (**Figure 5D**). Further verification by qRT-PCR showed that the expression of MYBL2 in 40 LUAD tissue samples was significantly lower than that in the adjacent tissue (**Figure 5E**). In addition, the correlation analysis of the expression levels of LINC00346 and MYBL2 in LUAD tissue showed that the expression levels of LINC00346 and MYBL2 were highly positively correlated (**Figure 5F**). The survival rate of patients with high MYBL2 expression was significantly lower than that with low expression (**Figure 5G**). Clinical stage analysis showed that the expression level of MYBL2 showed significant differences in different clinical stages and N stage (**Figure 5H**). The targeting sites of MYBL2 and miR-30c-2-3p were predicted by TargetScan (**Figure 5I**), and their binding relationship was verified by dual luciferase and RIP assays. Compared with the control group, overexpression of miR-30c-2-3p significantly reduced the luciferase activity of A549 cells co-transfected with MYBL2-wt while no obvious change was found in A549 cells co-transfected with MYBL2-mut (**Figure 5J**). RIP results showed that after dropping-down Ago2 protein, MYBL2 enriched significantly in the immunoprecipitation with Ago2 in miR-30c-2-3p mimic group (**Figure 5K**), indicating that MYBL2 could directly bind to miR-30c-2-3p in NCI-H1395 and A549 cells. Moreover, the results of qRT-PCR and western blot indicated that knockdown of LINC00346 increased the expression level of miR-30c-2-3p, while reduced the mRNA and protein levels of MYBL2. Inhibiting miR-30c-2-3p or overexpressing MYBL2 partially reversed the effects of knocking down LINC00346 on MYBL2 expression level (**Figures 5L, M** **)**. In summary, the above results indicated that LINC00346 regulates the expression of MYBL2 in LUAD cells by sponging miR-30c-2-3p.

CCNA2 has been reported to affect the cell cycle of various cancers (17, 18). We found that LINC00346, miR-30c-2-3p and MYBL2 were significantly enriched in the CELL CYCLE signaling pathway through GSEA enrichment analysis (**Figures S1A–C**). Then, we demonstrated that whether LINC00346 regulated the CELL CYCLE signaling pathway through the miR-30c-2-3p/MYBL2 axis. Western blot was used to detect the expression of CELL CYCLE signaling pathway related proteins, and the results were shown in **Figure 5M**. Inhibition of LINC00346 reduced the expression of cyclin B1, cyclin A2 and Plk1, while the effects were abolished by inhibition of miR-30c-2-3p or overexpression of MYBL2. So, LINC00346 could regulate the CELL CYCLE signaling pathway by sponging miR-30c-2-3p and targeting MYBL2.

### LINC00346 Acts as a ceRNA and Regulates the Growth of LUAD Cells by miR-30c-2-3p and Targeting MYBL2

**Figure 6:**
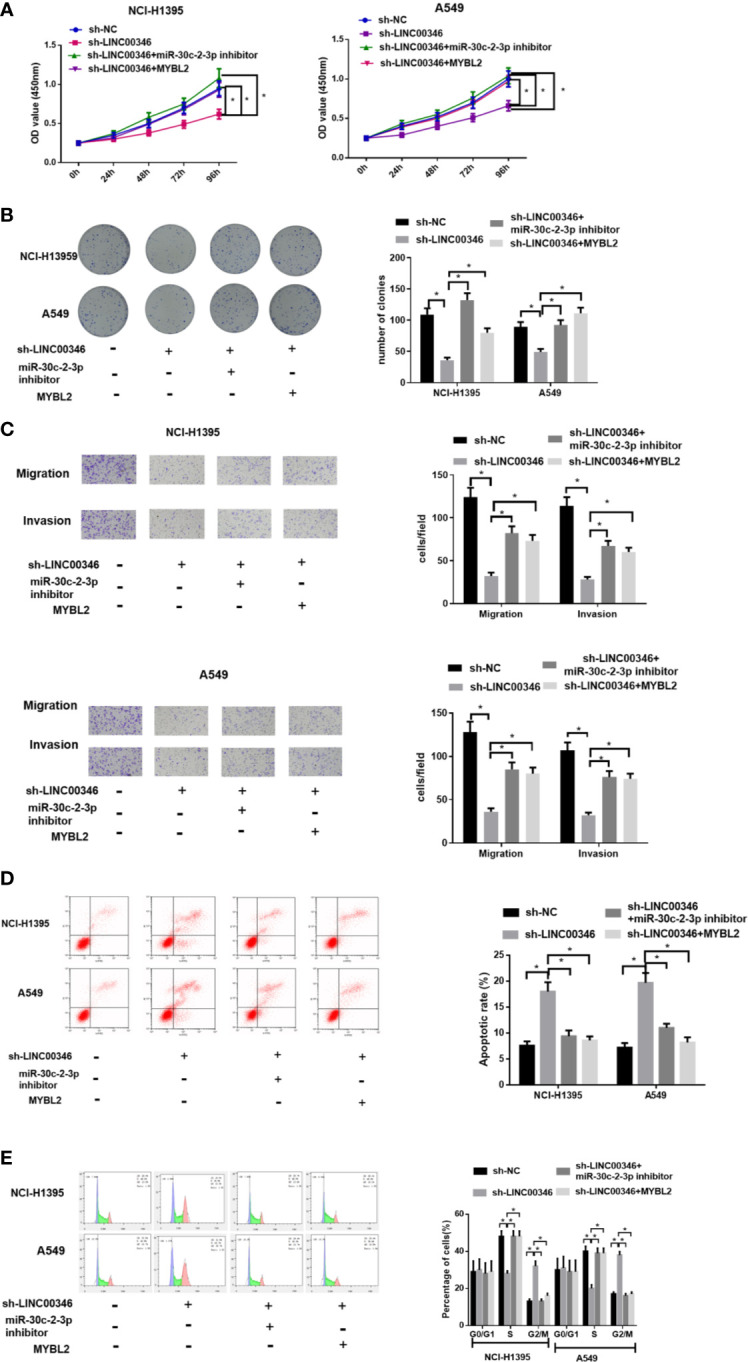
LINC00346 promotes the growth of LUAD cells by regulating the miR-30c-2-3p/MYBL2 axis. **(A)** Cell proliferation, **(B)** colony formation ability, **(C)** migration and invasion (100×), **(D)** apoptosis and **(E)** cell cycle progression of NCI-H1395 and A549 cells were detected by CCK-8, colony formation, Transwell and FCM assays, respectively. *P < 0.05.

In order to investigate whether LINC00346 plays its carcinogenic role by sponging miR-30c-2-3p and targeting MYBL2, we set up sh-LINC00346 group, sh-LINC00346+miR-30c-2-3p inhibitor group, and sh-LINC00346+MYBL2 group. The results showed that knockdown of LINC00346 inhibited the proliferation, migration and invasion, promoted cell apoptosis, and slowed down the cell cycle process of NCI-H1395 and A549 cells. Inhibition of miR-30c-2-3p or overexpression of MYBL2 could reverse the effects of LINC00346 on NCI-H1395 and A549 cells (**Figures 6A–E**). These findings suggested that LINC00346 promoted the development of LUAD by regulating the miR-30c-2-3p/MYBL2 axis.

### LINC00346 Knockdown Inhibits LUAD Tumor Growth *In Vivo*

**Figure 7:**
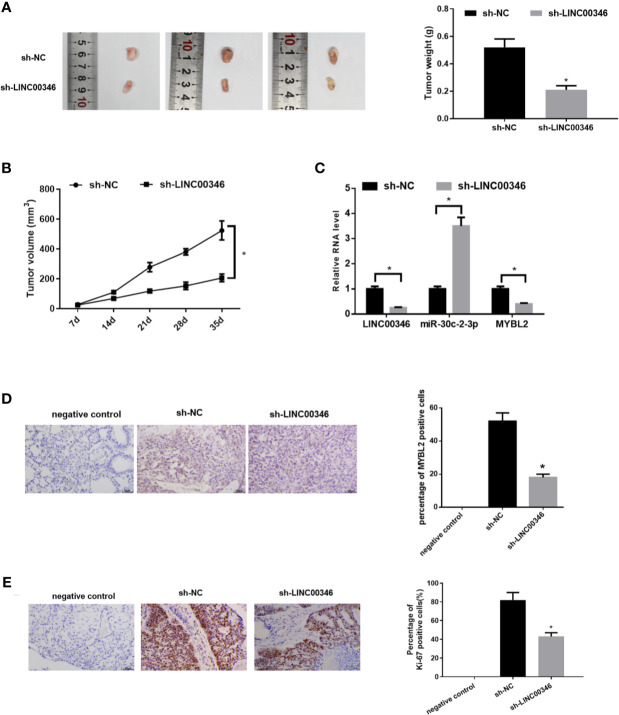
Knockdown of LINC00346 inhibits the growth of LUAD. **(A)** Xenograft tumors were photographed and tumor weight was measured from nude mice after 35 d; **(B)** Tumor volume was measured weekly; **(C)** The expressions of LINC00346, miR-30c-2-3p and MYBL2 in tumor tissues were determined by qRT-PCR; **(D, E)** IHC detected the number of Ki-67 positive cells in xenograft tumor tissues (400×). *P < 0.05.

To further analyze the effects of LINC00346 on LUAD, we established mouse xenograft tumor models *in vivo*. After 35 days, we found that compared with sh-NC group, the tumor volume and weight of sh-LINC00346 group were significantly reduced (**Figures 7A, B** **)**. Then we evaluated the expressions of LINC00346, miR-30c-2-3p and MYBL2 in tumor tissues by qRT-PCR. The results exhibited that, compared with the control group, the expressions of LINC00346 and MYBL2 were lower and expression of miR-30c-2-3p was higher in sh-LINC00346 group (**Figure 7C**). MYBL2 and Ki-67 immunostaining showed that there were significantly fewer MYBL2 and Ki-67 positive cells in subcutaneous tumors in A549 cells with knockdown of LINC00346 (**Figures 7D, E**). These results suggested that LINC00346 knockdown inhibited LUAD tumor growth *in vivo*.

## Discussion

With the development of human transcriptome sequencing, some lncRNAs related to the development of LUAD have been identified. Except for the feature lncRNAs, the potential necessary lncRNAs and their functions along with regulatory mechanisms in regulating LUAD are still worth studying. Herein, we identified the up-regulated LINC00346 in LUAD based on gene expression profile analysis, and studied the effects of LINC00346 knockdown on the biological function of LUAD cells through *in vitro* and *in vivo* experiments. It was found that LINC00346 knockdown inhibited the proliferation, migration and invasion of LUAD cells and promoted apoptosis by blocking the cell cycle in G2/M phase. This is consistent with the studies on bladder cancer (15) and gastric cancer (19). *In vivo* experiments observed that the knockdown of LINC00346 inhibited the tumor growth of LUAD, indicating that LINC00346 functioned as an oncogene.

The ceRNA hypothesis presents that mRNA and lncRNA can regulate each other through common miRNA response element (20). LINC00346 can act as a sponge of miRNA to play its regulatory role in a variety of cancers. For example, LINC00346 may regulate the expression of WDR18 through competitively binding to miR-542-3p and promote the development of HCC (13). LINC00346 promotes pancreatic cancer growth and gemcitabine resistance by sponging miR-188-3p and regulating expression of BRD4 (21). In this study, in order to find the downstream miRNA of LINC00346 in LUAD, we conducted bioinformatics analysis, dual-luciferase reporter gene and RIP assays, and determined that LINC00346 was the molecular sponge of miR-30c-2-3p. We further confirmed that inhibition of miR-30c-2-3p could reverse the effects of LINC00346 knockdown on LUAD cell proliferation, migration, invasion and cell cycle progression. Compared with published literature, this study highlights the role of LINC00346 in LUAD and its downstream mechanism affecting LUAD progression, which has not been previously reported. Similarly, the role of LINC00346 is similar to that previously reported, and LINC00346 still plays a role as an oncogenic gene in LUAD. The molecular mechanism of miR-30c-2-3p in gastric cancer and breast cancer has been reported. In gastric cancer, miR-30c-2-3p participates in RAB31/GLI1 signal transduction by targeting RAB31 (22). miR‐30c‐2‐3p negatively regulates NF‐кB signaling by targeting TRADD in breast cancer cells (23). Additionally, although Namhee Yu et al. (24) identified the prognostic value of miR-30c-2-3p in LUAD by sequencing, the regulatory mechanism of this miRNA in LUAD has not been reported.

The article found that MYBL2 was a downstream target of miR‐30c‐2‐3p and was highly expressed in LUAD tissues with the capable of predicting a poor prognosis, which was consistent with the studies in esophageal squamous cell carcinoma (25), HCC (26) and pancreatic ductal carcinoma (27). We further verified the relationship among LINC00346, miR-30c-2-3p and MYBL2, and found that LINC00346 regulated the expression of MYBL2 in LUAD cells by sponging miR-30c-2-3p. Besides, we conducted GSEA pathway enrichment analysis to prove that LINC00346, miR-30c-2-3p and MYBL2 were enriched in the CELL CYCLE pathway. Similar to previous studies, MYBL2 can regulate tumor progression by regulating CELL CYCLE signaling pathway in esophageal squamous cell carcinoma and colorectal cancer (25, 28). We then confirmed these predictions through cellular and molecular experiments. The inhibition of miR-30c-2-3p or overexpression of MYBL2 could reverse the inhibitory effects of LINC00346 knockdown on the expressions of CELL CYCLE signaling pathway related proteins cyclin B1, cyclin A2 and Plk1 as detected by qRT-PCR and western blot, indicating that LINC00346 regulated CELL CYCLE signaling pathway by targeting miR-30c‐2‐3p/MYBL2 axis. The regulatory effect of this mechanism on cell malignant behaviors was verified by rescue experiments, and it was found that inhibition of miR-30c-2-3p or overexpression of MYBL2 could reverse the effects of sh-LINC00346 on the development of NCI-H1395 and A549 cells. LINC00346 carries the “seed sequence” of miR-30c-2-3p and can adsorb miR-30c-2-3p like a sponge, thus reducing the binding of miRNA and its downstream target gene MYBL2 and preventing the degradation of MYBL2 by miR-30c-2-3p. Furthermore, the CELL CYCLE signaling pathway is regulated to promote the malignant progression of LUAD.

**Figure 8:**
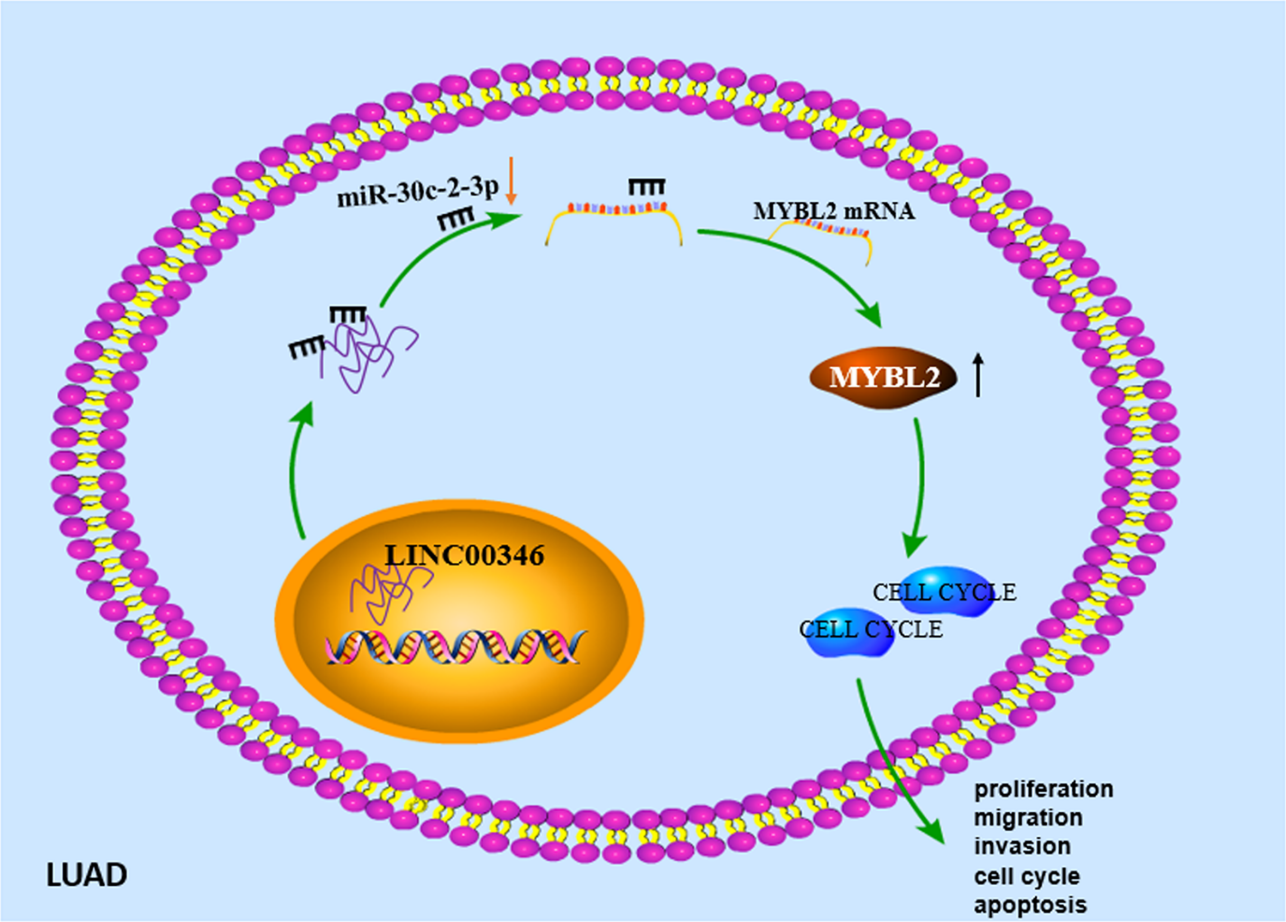
Molecular mechanism diagram of LINC00346/miR-30c-2-3p/MYBL2 axis regulating cell cycle signaling pathway to promote malignant progression of LUAD.

In summary, we determined that LINC00346 played an important role as an oncogene in LUAD. Our study confirmed for the first time that LINC00346/miR-30c-2-3/MYBL2 axis regulated the CELL CYCLE signaling pathway and promoted the development of LUAD (**Figure 8**). This regulatory network may help to elucidate the tumorigenesis of LUAD and may provide theoretical basis for the development of new diagnostic and therapeutic methods for LUAD. However, there are still some deficiencies. In the future, we will further analyze the correlation between the above gene expression and the prognosis and clinicopathology of LUAD patients, so as to further explain their prognostic or diagnostic value in LUAD.

## Data Availability Statement

The original contributions presented in the study are included in the article/**Supplementary Material**. Further inquiries can be directed to the corresponding author.

## Ethics Statement

The patients signed informed consent. The project was approved by the research ethics committee of Hangzhou Red Cross Hospital.

## Author Contributions

All authors listed have made a substantial, direct, and intellectual contribution to the work, and approved it for publication.

## Conflict of Interest

The authors declare that the research was conducted in the absence of any commercial or financial relationships that could be construed as a potential conflict of interest.

## Publisher’s Note

All claims expressed in this article are solely those of the authors and do not necessarily represent those of their affiliated organizations, or those of the publisher, the editors and the reviewers. Any product that may be evaluated in this article, or claim that may be made by its manufacturer, is not guaranteed or endorsed by the publisher.

## Supplementary Material

Supplementary Figure 1 GSEA pathway enrichment analysis results of **(A)** LINC00346, **(B)** miR-30c-2-3p and **(C)** MYBL2.
