# Supplementary figures and images for: LINC00346 Sponges miR-30c-2-3p to Promote the Development of Lung Adenocarcinoma by Targeting MYBL2 and Regulating CELL CYCLE Signaling Pathway
Source: Front Oncol. 2021 Sep 22;11:687208. doi: 10.3389/fonc.2021.687208 (PMC8493815; doi:10.3389/fonc.2021.687208)

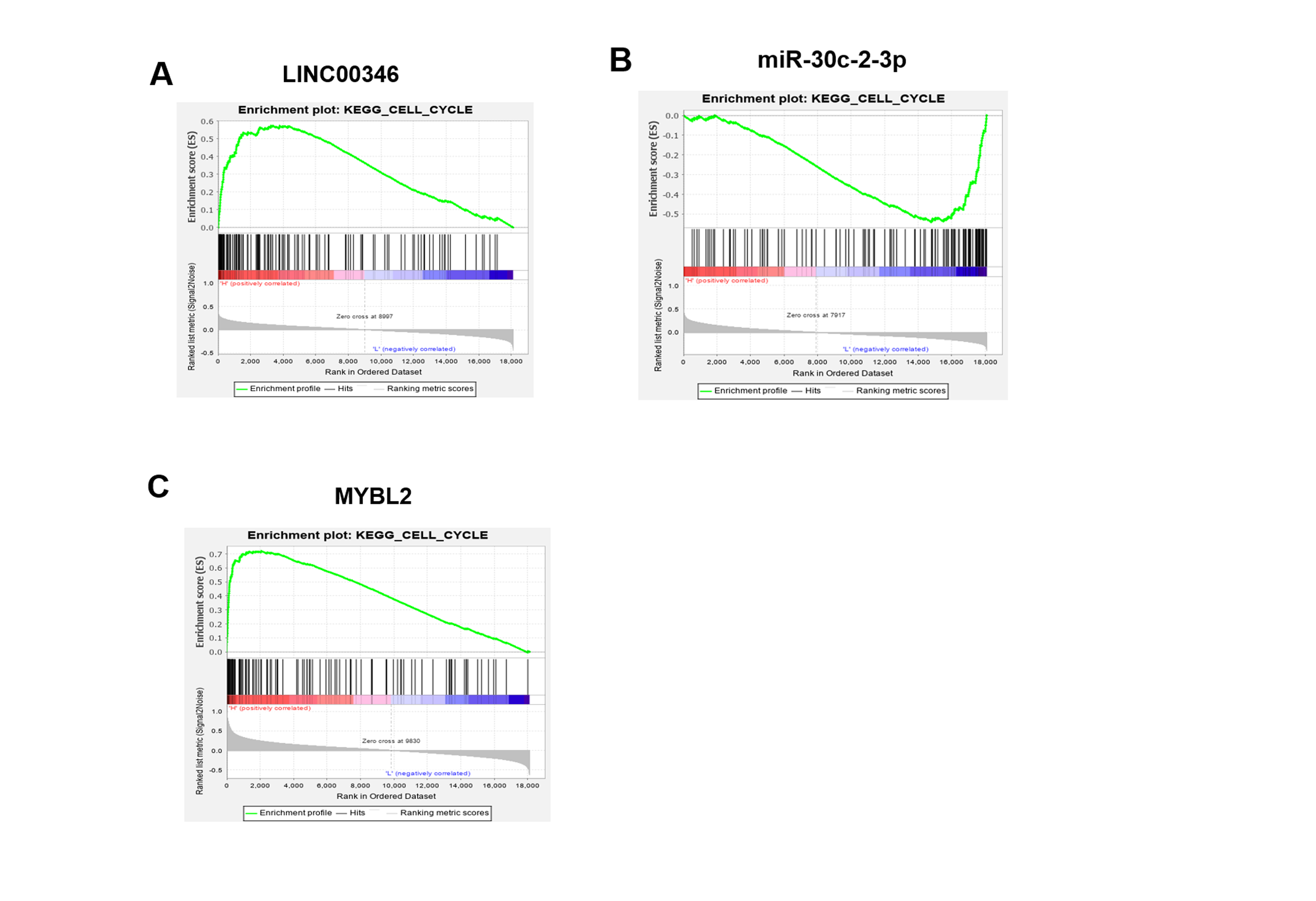

Supplement: Supplementary Figure 1 — GSEA pathway enrichment analysis results of (A) LINC00346, (B) miR-30c-2-3p and (C) MYBL2. [file Image_1.tif]
